# Chemotaxonomic Study of *Citrus*, *Poncirus* and *Fortunella* Genotypes Based on Peel Oil Volatile Compounds - Deciphering the Genetic Origin of Mangshanyegan (*Citrus nobilis* Lauriro)

**DOI:** 10.1371/journal.pone.0058411

**Published:** 2013-03-13

**Authors:** Cuihua Liu, Dong Jiang, Yunjiang Cheng, Xiuxin Deng, Feng Chen, Liu Fang, Zhaocheng Ma, Juan Xu

**Affiliations:** 1 Key Laboratory of Horticultural Plant Biology (Ministry of Education), National Key Laboratory of Crop Genetic Improvement, College of Horticulture and Forestry, Huazhong Agricultural University, Wuhan, People's Republic of China; 2 Citrus Research Institute, Chinese Academy of Agricultural Sciences, Chongqing, People's Republic of China; 3 Department of Food, Nutrition and Packaging Sciences, Clemson University, Clemson, South Carolina, United States of America; China Agricultural University, China

## Abstract

Volatile profiles yielded from gas chromatography-mass spectrometry (GC-MS) analysis provide abundant information not only for metabolism-related research, but also for chemotaxonomy. To study the chemotaxonomy of Mangshanyegan, its volatile profiles of fruit and leaf and those of 29 other genotypes of *Citrus*, *Poncirus*, and *Fortunella* were subjected to phylogenetic analyses. Results showed that 145 identified (including 64 tentatively identified) and 15 unidentified volatile compounds were detected from their peel oils. The phylogenetic analysis of peel oils based on hierarchical cluster analysis (HCA) demonstrated a good agreement with the Swingle taxonomy system, in which the three genera of *Citrus*, *Poncirus*, and *Fortunella* were almost completely separated. As to *Citrus*, HCA indicated that Citrophorum, Cephalocitrus, and Sinocitrus fell into three subgroups, respectively. Also, it revealed that Mangshanyegan contain volatile compounds similar to those from pummelo, though it is genetically believed to be a mandarin. These results were further supported by the principal component analysis of the peel oils and the HCA results of volatile profiles of leaves in the study.

## Introduction

Germplasm research provides clues to the origination, development, and even utilization of a biological material and is a prerequisite to collect and protect core collection of plant genetic resources. Information about individual accessions, particularly those found *in situ*, is often poor, reducing the frequency and efficiency of utilization and the ultimate benefits [1]. Recently, there is growing recognition that the germplasm diversity affects agricultural development, food security, livelihoods, and development aspirations of every country. The collection, preservation, and evaluation of germplasm are of great importance to the world citrus industry [2], [3].

Mangshanyegan (*Citrus nobilis* Lauriro), a wild germplasm in the citrus family, was discovered about 30 years ago in the remote mountainous forests of Mangshan, Hunan Province, China [4]. So far two genotypes of round- and sharp-leaf type Mangshanyegan have been found, whereas they have similar fruit types and their fruits can send forth a pleasant and intensive balsamic and floral aroma. Just like in other genotypes of *Citrus* and its relatives, monoterpenoids and sesquiterpenoids play dominant roles in the volatile profile of Mangshanyegan peel oil. Additionally, acids, alcohols, aldehydes, esters, an alkane, an indole, and a diterpene were all identified [5]. Thus, Mangshanyegan is not only a promising and precious resource for the essential oil industry, but also a desirable object for researches regarding mechanisms of aroma production. However, its genetic origination remains uncertain. Therefore, volatile compounds are ideal objects that can provide abundant information for chemotaxonomy study.

In chemotaxonomy study, the chromatographic identification of either volatile or non-volatile natural compounds was a tedious lab work. Recently, progress in chromatographic/spectral technique and software for automatically analyzing MS data, such as MzMine [6], MathDAMP [7], Tagfinder [8] and MetAlign [9], has remarkably facilitated chemotaxonomy and nontargeted functional genomics research [10], [11]. The former would play an important role in the study of taxonomy and has been used in chemotaxonomy studies of fungi and plants, and is a promising method for those highly hybrid plants and its closely related species, such as *Citrus* and its related genera [12]–[14].

As reviewed by Moore [15], *Citrus* and its relatives have some distinguishing characteristics: (1) *Citrus* and its relatives are very ancient, which makes it difficult to trace them back to their origins and diversities; (2) *Citrus* and its relatives are highly heterozygous and include many hybrids; (3) polyembryo occurs in most *Citrus* and its relatives, and it's very possible that nucellar embryos triumph over the single zygotic embryo. The items mentioned above make traditional morphology and geography insufficient to clarify their taxonomy, so various biochemical and molecular markers were used to solve the problem. Since Kesterson et al. [16] and Pieringer et al. [17] analyzed the volatile constituents of different citrus leaf oils in 1964, the chemotaxonomy in *Citrus* and its related genera have been widely reported [18]–[20].

Luro et al. [21] studied the genetic diversity and chemical diversity among 24 citron varieties (*C. medica* L.) based on 22 nuclear and 6 cytoplasmic genetic markers along with 43 volatile compounds identified from leaf essential oils. The authors found that the diversity based on leaf oil compositions did not agree with the molecular diversity and was unsuitable for intraspecific phylogenetic studies. However, chemotaxonomy studies on other plants showed that chemical compounds (e.g., Sesquiterpene dialdehyde, etc) could be considered as species markers [22]. Furthermore, Hou et al. [23] found that chemotaxonomic classification could be very useful for aquatic assessment in distinguishing phytoplankton communities and extremely advantageous and cost-effective in large ecosystem-scale research. Li et al. [24] found that the evolution and classification of bamboos inferred from leaf wax n-alkanes were consistent with morphological investigations reported previously. These above studies suggested that chemotaxonomic analysis was a reliable, informative, high-throughput research tool for taxonomy study. Also, it is well known that wild and primitive genotypes, with higher genetic diversity, were significant to the taxonomic classification study [25], [26]. Thus, the phylogeny of Mangshanyegan and the chemotaxonomy of *Citrus* and its related genera were assessed in this study based on volatile compounds of their peel oils and volatile profiles from their leaves, which may supply both some new clues to the evolution of citrus and detailed information of the resolved volatile compounds of peel oil for further use, such as in cosmetic industry or citrus breeding programs on sensory flavor quality.

## Materials and Methods

### Plant material

Including the sharp- and round-leaf genotypes of Mangshanyegan, 30 genotypes of mature fruits belonging to three genera of *Citrus*, *Poncirus*, and *Fortunella* were collected from National Citrus Breeding Centre of China (NCBC), Wuhan in Hubei province, or Citrus Research Institute of Chinese Academy of Agricultural Sciences, Chongqing (CRIC) in 2009 or 2010. Only one sample, Shatian pummelo, was collected from Citrus Experimental Station (CES), Zigui county, Hubei Province. All necessary permits were obtained for this study. Xiuxin Deng (the authority for NCBC), Dong Jiang (for CRIC), and Wenhua Song (for CES) granted the permission for utilization of samples from corresponding location.

Leaf samples of 29 genotypes collected from NCBC, CRIC, and CES were analyzed. Among them, 25 genotypes were corresponding to that of fruit samples. The mature spring-flush leaves on the third and forth nodes from the biological basal end were collected in July, 2010 and stored at −80°C until analysis.

The detailed information of the leaf and fruit samples was listed in **Table 1**.

**Table 1:** Citrus genotypes used in this study.

Abbreviation*	Common name	Scientific Name	Sampling location and time
**Early**	**early-flowering type trifoliate**	***Poncirus trifoliata*** **L. Raf.**	**NCBC a, 2009**
Trifoliate	Trifoliate orange	*Poncirus trifoliata* L. Raf.	NCBC, 2009
Kumquat-HZAU	Hongkong kumquat	*Fortunella hindsii* Swingle	NCBC, 2009
Kumquat-CRIC	Hongkong kumquat	*Fortunella hindsii* Swingle	CRIC b, 2010
*Ningbo*	*Ningbo Meiwa Kumquat*	*Fortunella crassifolia* Swingle	NCBC, 2010
**Calamondin**	**Calamondin**	***C. madurensis*** **Lour.**	**NCBC, 2009**
*Sanshan Xiangyuan*	*Sanshan*	*C. wilsonii* Tanaka L.	CRIC, 2010
Yuzu	Yuzu	*C. junos* Sieb.	CRIC, 2010
FC	Finger citron	*C. medica* L.	NCBC, 2010
Eureka^,^	Euroka lemon	*C. limon* (L.) Burm. f.	NCBC, 2009
Limonera	Limonera lemon	*C. limon* (L.) Burm. f.	CRIC, 2010
Rough	Rough lemon	*C. jambhiri* (L.) Lush	NCBC, 2009
Lime	Lime	*C. aurantifolia* Swing.	NCBC, 2009
**Ichang**	**Ichang papeda**	***C.ichangensis*** **Swing.**	NCBC, 2010
*HP*	*Honghe papeda*	*C. honghensis.* Y. L. D. L	CRIC, 2010
Liang	Liangping pummelo	*C. grandis* Osbeck	CRIC, 2010
Kaopan	Kaopan pummelo	*C. grandis* Osbeck	NCBC, 2010
Shatian	Shatian pummelo	*C. grandis* Osbeck	CES^c^, 2009
Tachibana	Tachibana orange	*C. tachibana* Makino	CRIC, 2010
**Mang-HZAU-09**	**Mangshanyegan (sharp leaf)**	***C. nobilis*** **Lauriro**	**NCBC, 2009**
Mang-HZAU	Mangshanyegan (sharp leaf)	*C. nobilis* Lauriro	NCBC, 2010
Mang-SL-CRIC	Mangshanyegan (sharp leaf)	*C. nobilis* Lauriro	CRIC, 2010
Mang-RL-CRIC	Mangshanyegan (round leaf)	*C. nobilis* Lauriro	CRIC, 2010
Kaime	Kamei satsuma mandarin	*C. unshiu* Marcow	NCBC, 2010
Dao	Daoxian wild mandarin	*C. reticulata* Blanco	NCBC, 2010
Jiang	Jiangyong wild mandarin	*C. reticulata* Blanco	NCBC, 2010
Cha	Chazhigan mandarin	*C. reticulata* Blanco	CRIC, 2010
Ponkan	Ponkan mandarin	*C. reticulata* Blanco	NCBC, 2010
**Huangyan**	**Huangyanbendizao tangerine**	***C. reticulata*** **Blanco**	**NCBC, 2009**
Hua	Huanongbendizao tangerine	*C. reticulata* Blanco	NCBC, 2009
Nanfeng	Nanfengmiju mandarin	*C. reticulata* Blanco	NCBC, 2009
Clementine	Clementine tangerine	*C. reticulata* Blanco	NCBC, 2009
*Mang-T*	*Mangshan wild tangerine*	*C. reticulata* Blanco	NCBC, 2010
Seike	Seike navel orange	*C. sinensis* Osbeck	NCBC, 2010

a Collected from National Citrus Breeding Centre of China (NCBC).

b Collected from Citrus Research Institute of Chinese Academy of Agricultural Sciences (CRIC).

c Collected from Citrus Experimental Station (CES), Zigui county, Hubei Province, China.

* The samples in bold were collected only for peel oils analysis, while the samples in italic were collected only for leaf volatile analysis, and the samples in normal font were for both analyses. The abbreviations of fruit samples belonging to Sinocitrus were marked with underline.

### Standards and reagents

Internal standards of chlorononane and methyl nonanoate were obtained from Sigma Co. Ltd (St Louis, MO, USA). A standard series of C_7_–C_30_ saturated alkanes bought from Supelco (Bellefonte, PA., USA) was used for retention index determination. Methyl tert-butyl ether (MTBE) (high performance liquid chromatography grade) from Tedia (Fairfield, USA) was applied to extracting volatiles. Anhydrous sodium sulfate was purchased from Sinopharm Chemical Reagent Co., Ltd (Shanghai, China). The sources of volatile standards are listed in **Table S1**.

### Extraction and volatile analysis of the peel oil

The sample preparation and the volatile extraction by solvent MTBE were conducted according to Liu et al. [5]. Three independent biological replicates were prepared. Three grams of fruit peel were used for the volatile extraction with 15 mL MTBE. Then 8697 µg of chlorononane and 400 µg of methyl nonanoate were added as the internal standards. After 1 h of microwave assisted extraction using an FS60 ultrasonic cleaner (Fisher Scientific, Pittsburgh, PA, USA), the organic layer was collected, dried over Na_2_SO_4_ and concentrated to a final volume of 1.4 mL under a gentle stream of nitrogen.

The extract was analyzed using a TRACE GC Ultra GC coupled to a DSQ II mass spectrometer (Thermo Fisher Scientific, Waltham, MA, USA) and equipped with a TRACE TR-5 MS column (30 m×0.25 mm×0.25 µm, Thermo Scientific, Bellefonte, PA, USA). The parameters of gas chromatography and mass spectrometer were set according to the method described by Liu et al. [5]. Helium was used as the carrier gas, with a split ratio of 50∶1, at a flow rate of 1 mL/min. The temperatures of the injection port, ion source, and MS transfer line were kept at 250, 260, and 280°C, respectively. The oven temperature program adopted the following procedure, which started from 40°C for the initial 3 min, then increased to 160°C at a rate of 3°C/min, kept at 160°C for 1 min, then followed by a ramp of 5°C/min to reach 200 °C, held for 1 min, raised to 240°C at a rate of 8°C/min, and finally kept at 240°C for 3 min. The MS was collected in a positive electron ionization mode at 70 eV, obtaining spectra with a scan range from *m*/*z* 45 to 400.

The raw data obtained from GC-MS were processed with Xcalibur and AMDIS software. The volatile compounds were identified on the basis of the NIST/EPA/NIH Mass Spectral Library (NIST 2008) and Wiley Registry of Mass Spectral Data 8th edition. Retention indexes were calculated with a homologous series of *n*-alkanes (C_7_–C_30_) [27]. Eighty-one volatile compounds were further positively identified based on the authentic standards (listed in **Table S1**).

### Solid phase microextraction (SPME) analysis for leaves

After being ground in liquid nitrogen, 2 g of the finely powdered leaf samples was transferred into a 20 mL Teflon cap vial (Thermo Fisher Scientific) with 5 mL of NaCl aqueous solution (25%, w/v) being added later. Then, the vial was sealed tightly. The extraction procedure described by Liu et al. [28] was applied with minor modifications. The sealed vial was incubated at 40°C for 30 min and the extraction of volatile compounds with a 2 cm, 50/30 µm carboxen divinylbenzene polydimethylsiloxane (CAR/DVB/PDMS) Supelco SPME fiber (Sigma-Aldrich Co. Ltd., St. Louis, MO, USA) was conducted at 40°C for 45 min by agitation at 10 sec intervals. The volatiles were desorbed from the SPME fiber at 230°C for 1 min in the injection port. After each extraction, the fiber was conditioned at 240°C for 3 min. Three replicates for every leaf sample were prepared.

### Data analysis

For volatile compounds of the peel oils, the peak areas in the total ion current chromatogram (TIC) were processed by the software of Xcalibur. The corrected peak areas (CPAs) of target compound were calculated based on internal standards. Chlorononane was used to calculate the CPA of *β*-myrcene and *d-*limonene, and methyl nonanoate was used for all the other volatile compounds. At first, when each peak area of the internal standards (chlorononane and methyl nonanoate) in Kaime satsuma mandarin was set as 1 for calculating CPAs of different volatile compounds, the chromatographic peak area of each corresponding internal standard in every sample was normalized respectively. Then, every peak area of targeted volatile compounds was divided by a corresponding CPA of the internal standard in every sample. The result was named as the corrected peak area of target compound (CPA-TC), which was used for Hierarchical Cluster Analysis (HCA) and Principal Component Analysis (PCA).

For HCA, the CPA-TCs were transformed via log 2 with the MultiExperiment Viewer (MeV) version 4.7.4 software (http://www.tm4.org, Dana-Farber Cancer Institute, Harvard Medical School, Boston, MA, USA). The average linkage clustering was performed based on the Pearson correlation [10]. The complete dataset including all replicates was employed for HCA, whereas only the mean values of volatile compounds in each sample were used for PCA. After autoscaling pretreatment with CPA-TCs was done as van den Berg et al. [29], the functions of Prcomp and Plot in R version 2.14.2 software (http://www.R-project.org, R Development Core Team) were employed for PCA. The raw dataset of leaf volatile profiles was preprocessed according to non-targeted method with Metalign software Package (version 200410, http://www.metalign.nl, Plant Research International, Wageningen, The Netherlands) referring to Lommen [9] and Tikunov et al. [10], and then the preprocessed result (**Table S2**) was subjected to HCA using MeV based on Cosine correlation and single linkage method.

## Results

### Volatile compounds detected in peel oils

In this study, a total amount of 160 volatile compounds were detected in the peel oils (**Table S1**), among which 81 were definitely identified, 64 tentatively identified, and 15 unidentified. The above 145 identified compounds were grouped into the following 19 classes: acids (3 compounds), alcohols (9), aldehydes (11), alkane (1), diterpene (1), esters (6), furans (2), monoterpenes (16), monoterpene alcohols (15), monoterpene aldehydes (4), monoterpene esters (8), monoterpene ketones (4), monoterpene oxides (4), sesquiterpenes (36), sesquiterpene alcohols (11), sesquiterpene aldehydes (2), sesquiterpene ketone (1), sesquiterpene oxide (1), benzene compounds (10).

In this study, five novel compounds, (*E*)-3-caren-2-ol, *α*-copaene-11-ol, (*Z, E*)-α-Farnesene, *γ*-himachalene, and 8-isopropenyl-1,5-dimethyl-cyclodeca-1,5-diene were tentatively identified in citrus fruit for the first time. For Mangshanyegan, *α*-copaene-11-ol was found in peel oil of both genotypes, while 8-isopropenyl-1,5-dimethyl-cyclodeca-1,5-diene was detected only in the peel oil of sharp-leaf genotype (**Table S1**).

### HCA results of peel oils

In this study, the mass spectral data of volatile profiles supplied abundant information to the chemotaxonomy study of Mangshanyegan. HCA was conducted throughout 90 data sets of peel oils from all 30 samples, including the sharp-leaf and round-leaf Mangshanyegan collected from CRIC in 2010 and the sharp-leaf collected from NCBC in 2009 and 2010.

**Figure 1:**
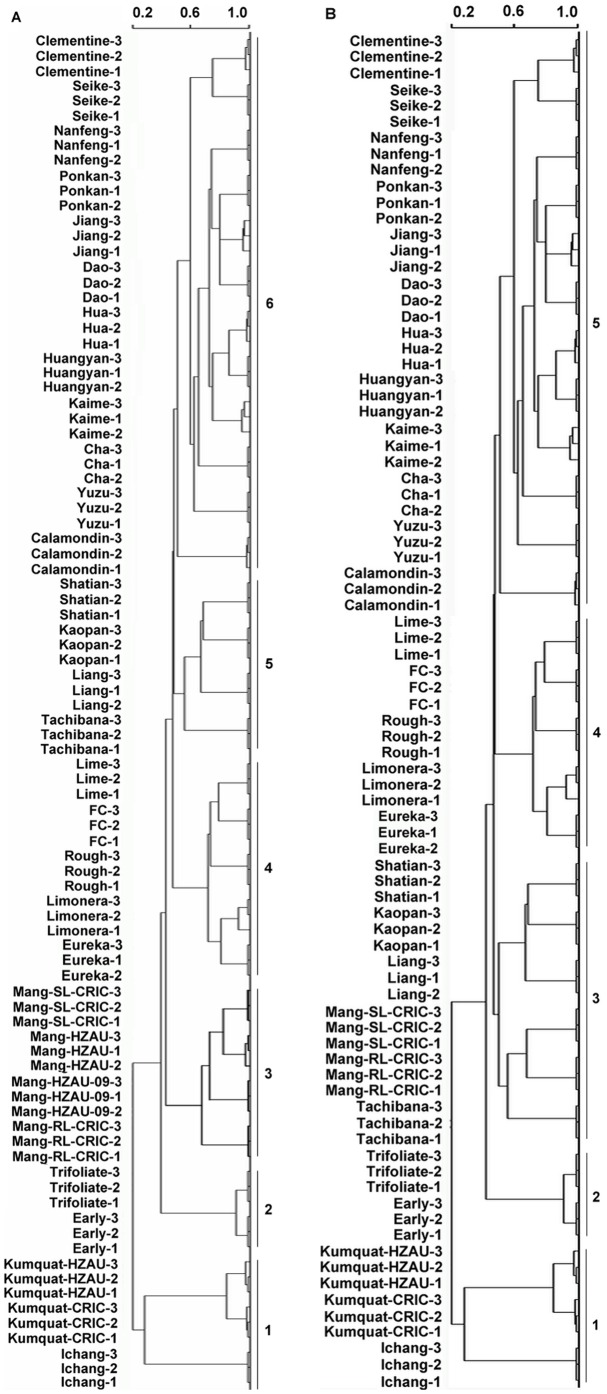
Hierarchical cluster analysis (HCA) results of peel oil samples. (A) The whole sample set. (B) Samples without Mangshanyegan collected from National Citrus Breeding Centre of China in both 2009 and 2010.

The HCA results indicated that these 30 genotypes could be clustered into 6 groups: Group 1: one *C. ichangensis* and two *Fortunella hindsii*; Group 2: two *Poncirus trifoliate*; Group 3: four *C. nobilis*; Group 4: two *C. limon*, one *C. jambhiri*, one *C. medica*, and one *C. aurantifolia*. Group 5: three *C. grandis* and one *C. tachibana*; Group 6: one *C. madurensis*, one *C. junos*, one *C. unshiu*, one *C.sinensis*, and eight *C. reticulata* (**Figure 1A**).

The genotypes in Group 3, 4, 5, and 6 all belonged to *Citrus* genus, and the samples of the other two genera in this study were found in Group 1 and 2, respectively. Group 4, 5, and 6 represented Citrophorum (citron), Cephalocitrus (pummelo), and Sinocitrus (mandarin), respectively. This result confirmed the hypothesis that *C. grandis* (pummelo), *C. medica* (citron), and *C. reticulate* (mandarin) were the three basic species of the cultivated *Citrus* [30]. Each group could be recognized as a citrus true species together with its hybrids or descendants except *C. tachibana* Makino, a wild species from Japan, which was located in the group of pummelo instead of mandarin, and postulated as an individual species in Swingle classification system [31].

Three genera used in this study were almost separated, which was in accordance with previous studies [30], [32], except for Group 1 which included two *Fortunella hindsii* genotypes and Ichang papeda that belonged to *Citrus* genus. Also, it is worthy to note that the taxonomy of Ichang papeda was still controversial (this will be discussed later). This cluster dendrogram showed that *Citrus* and its related genera fell into different groups and the group of *Fortunella hindsii* Swingle located the most distantly. Mangshanyegan fell into a different group from the other three recognized groups of true basic species of the cultivated *Citrus*, and these four groups were sharply distinguished from each other (**Figure 1A**).

In the group of Mangshanyegan, the sharp-leaf ones collected from NCBC and CRIC in 2010 were grouped together, which neighbored with the sharp-leaf one from NCBC in 2009, whereas the round-leaf Mangshanyegan harvested from CRIC in 2010 was distant from the three sharp-leaf ones (Group 3 in **Figure 1A**). The results obviously indicated that the geographical and temporal influences on HCA were less than that of the genetic factor, which agreed with Merle et al. [33] and suggested the experimental reliability of the HCA result.

In the analysis of HCA, four samples of Mangshanyegan were used (see **Table 1**), and two accessions of Hongkong kumquats (one from NCBC and the other from CRIC) were investigated. However, only one accession of other citrus and its relatives was utilized. Accordingly, the differences of the sample numbers may cause a weighted bias among different genotypes, and the independent group of Mangshanyegan might result from this bias in sampling, which blurred its genetic divergence among samples.

To minimize the bias due to samples size and maximize the sample size, the sharp-leaf and round-leaf Mangshanyegan collected from CRIC in 2010 were used for HCA. Thus, although only five groups were obtained, the cluster result was almost identical to that with only either one of them included, and was different from that with four (**Figure 1A**) or three Mangshanyegan samples (data not shown). It was shown that Mangshanyegan was merged with the pummelos, forming the new Group 3 in **Figure 1B**, in which Mangshanyegan neighbored with Tachibana. Notably, the other four groups were hardly changed between **Figure 1A** and **Figure 1B**.

### PCA results of peel oils

**Figure 2:**
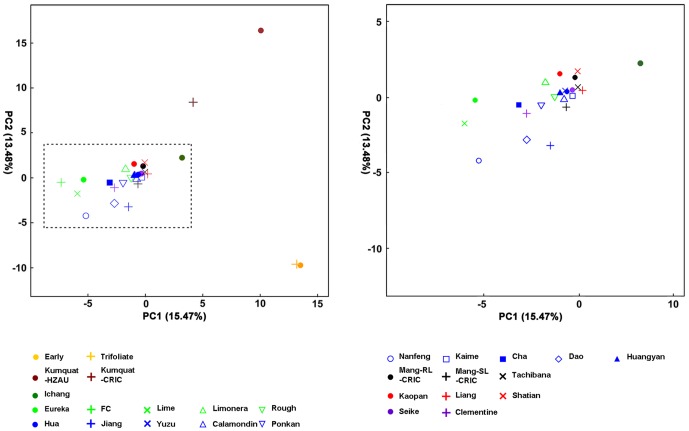
Principal Component Analysis (PCA) score plot of peel oils. (A) PCA graphic generated from principal component 1 (PC1) and 2 (PC2). (B) Partial PCA graphic within dash lines in (A).

PCA was applied to test the HCA results. The samples in each group in **Figure 1B** were colored individually (**Figure 2A** and **Figure 2B**). The first component explained 15.47% of the variance, and *Citrus* and *Poncirus* were clearly separated on the PC1 axis; the second component explained 13.84% of the variance, and all three genera used in the study were separated very clearly on the PC2 axis. Although the first two principal components explained only about 29% of the variance, three genera used in the study were distinguished from each other. Dots representing genotypes of *Poncirus* and *Citrus* were compact, whereas the two samples of *Fortunella* were scattered (**Figure 2B**). In general, the results of PCA were almost consistent with the results of HCA (**Figure 2B**).

### HCA results of leaf volatile profiles

**Figure 3:**
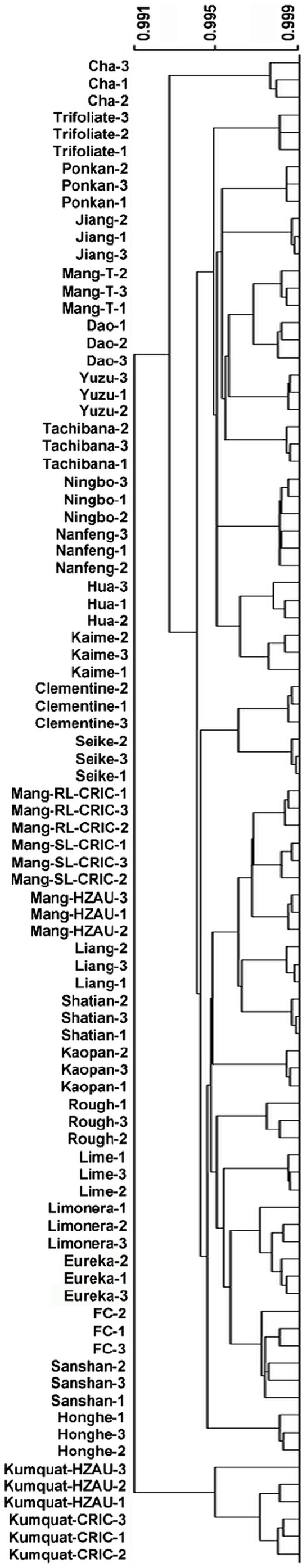
Hierarchical cluster analysis (HCA) results of leaf volatiles.

To verify the above results of HCA and PCA, the volatile compounds of leaf samples collected in 2010 were extracted by SPME and further analyzed with the assistance of MetAlign software. The aligned data obtained from MetAlign (**Table S2**) was subjected to MeV for HCA. The HCA results of leaf volatile profiles were almost in line with the results of HCA and PCA based on volatile compounds of peel oils. *C. tachibana* was clustered into the branch of mandarin, and Mangshanyegan was grouped with three pummelos (**Figure 3**).

Notably, Honghe papeda was clustered into the group of *Citrus* genus (**Figure 3**). Whereas, HCA of volatile compounds in peel oil demonstrated that the Ichang papeda fell into the group of *Fortunella* genus (**Figure 1A and 1B**).

## Discussion

### The origin of Mangshanyegan

Previous study speculated that Mangshanyegan (*Citrus nobilis* Lauriro) belongs to the King mandarin which might be a natural tangor (*C. reticulata* × *C. sinensis*) [32], whereas *C. sinensis* originated from the introgression of *C. reticulata* genotype with *C. grandis* [15], [34]. In the present study, **Figure 1B** and **Figure 3** showed that Mangshanyegan belonged to the group of pummelo instead of mandarin and neighbored with Tachibana (**Figure** **1B**) because the volatile profile of Mangshanyegan peel oil was similar to those of Tachibana (wild mandarin) and pummelo, indicating that Mangshanyegan is ancient and not pure mandarin genetic background. This is further supported by previous study as well. Li et al. [25] suggested that among 19 wild mandarin accessions and 33 loose-skin mandarin landraces, Mangshanyegan formed an individual group in the dendrograms constructed using nuclear simple sequence repeat (nSSR) and chloroplast simple sequence repeat (cpSSR) markers systems. In the nSSR tree, the Mangshanyegan group was the most distant one and very close to a mandarin landraces group (including Kuigan, Choupigan etc.), which was possible hybrids of mandarin with pummelo or with sweet orange [35]. And similar results were obtained except that the Kuigan group was the most distant group instead of Mangshanyegan group which was the second most distant group at the cpSSR loci [25].

However, it was reported that in the natural habitat of Mangshanyegan, there was neither any orange nor any pummelo found [36], [37]. Furthermore, it was reported that Mangshanyegan was a more primitive species than Mangshanyeju (*C. reticulata* blanco), and even might be the ancestor of Tachibana [36], [38]. Li and Liu [39] suggested that Mangshanyegan might be the intermediate type between the Ichang papeda and loose skinned mandarin. However, the above mentioned previous studies on Mangshanyegan were mainly based on morphology analysis and zymological analysis. The contradictions mentioned above could not be completely resolved in this study. An accurate conclusion will likely require genome resequencing.

In **Figure 1A**, Tachibana was clustered into the group of pummelo (Cluster 5) rather than the group of mandarin. In the Swingle's classification system, all mandarins and tangerines belonged to *C. reticulata* Blanco except Tachibana mandarin (*C. tachibana* Makino) and Indian wild orange (*C*. *indica* Tanakain). Based on the analyses of isozyme, chromosome, chloroplast DNA, and mitochondrial DNA, it was suggested that Tachibana was different from the mandarins originating from China and India, and it was separated from other mandarins at an early date [34], [40], [41]. According to our study, it could be concluded that *C. tachibana* Makino was distinct from other species of *Citrus* genera. However, previous reports never demonstrated that Tachibana was clustered with pummelo based on molecular markers [34], [42]–[44]. Thus, it was deduced that the possible reason for Tachibana falling into the cluster of pummelo in **Figure 1A** might be the uneven bias caused by the overwhelming sample number of Mangshanyegan, which emphasized the importance of population balance as the premise of chemotaxonomy. Possibly, owing to the much more similar volatile profile of peel oils between Tachibana and Mangshanyegan than that between Tachibana and other mandarins, Tachibana neighbored with Mangshanyegan in **Figure 1B**.

### Phylogenetic classification of other citrus

In **Figure 1A** and **Figure 1B**, the cluster dendrogram trees clearly showed that the cluster of *Fortunella hindsii* Swingle located most distantly. Previous studies suggested that *Fortunella* was the most primitive whereas *Citrus* was the most advanced genus among the “True Citrus Fruit Trees” [45], [46]. Based on the 11000 unigenes from a Clementine EST library, it was found that *Poncirus trifoliata* located in a cluster of citron-limes-lemon, whereas kumquat (*Fortunella japonica*) remained genetically distant to other citrus [47]. However, according to Barkley et al. [48], the group of *Poncirus* accessions was very distant from the four other citrus groups of mandarins, pummelos, citrons, and papedas, while kumquat was closer to the four citrus groups on the basis of genomic SSR. In this study, it should be noted that the volatile profiles of Kumquat was dominated by *β*-myrcene instead of *d*-limonene. The mean CPA-TC ratio between *β*-myrcene and *d*-limonene in Hongkong kumquat from NCBC and CRIC was about 192 and 206, respectively. Thus, the ratio was inferred as the predominant differences that caused the largest distance among cluster of Kumquat and other clusters in the HCA, as shown in **Figure 1**.

The taxonomy of *C. ichangensis* is controversial. With its flower resembling that of *Citrus* and its leaf resembling that of *Papeda*, *C. ichangensis* was grouped into the Papeda subgenus in Swingle's system [32]. However, in the Tanaka's system, it was classified into the subgenus Metacitrus [49]. The result of this study was in accordance with that of Handa et al. [50] and Nicolosi et al. [34]: *C. ichangensis*, which was distinct from the other samples of *Citrus* genus, located in the cluster of *Fortunella*.

By combining the previous morphological and biochemical criteria with molecular marker (RAPD, RFLP, and SCARs) analyses, Biswas et al. [51] supposed that Papeda was different from other *Citrus* species. Furthermore, Biswas et al. [52] found that Ichang papeda fell into the kumquat sub-cluster with 25 randomly selected SSR primer pairs among 40 species of *Citrus* and its related genera. In this study, Honghe papeda was close to *Citrus* genus while Ichang papeda was close to *Fortunella* genus. However, only the fruit peel of *C. ichangensis* and leaf of Honghe papeda were sampled, respectively. Therefore it needs more study in the same sample set.

In **Figure 1A** and **Figure 1B** **,** Chazhigan mandarin fell into the cluster of mandarin, while in **Figure 3**, Chazhigan mandarin was more distant from the other clusters of *Citrus* genus than the cluster of *Poncirus*. Volatile profile of Chazhigan mandarin revealed that the most abundant and predominant compound of its leaf was dimethyl anthranilate rather than *d*-limonene (**Figure S1**), which might be one of the reasons for its divergence from other genotypes in the *Citrus* genus in **Figure 3**.

### Validity of chemotaxonomic analysis for interspecies phylogenetic studies

The cluster results in this study agreed with the Swingle classification system very well. Luro et al. [47] also obtained a good agreement of diversity relationships with the established taxonomy and phylogeny among the species of citrus and its related genera based on EST-SSR markers. In addition, with 24 citron varieties employed in their study, Luro et al. [21] suggested that the diversity estimated by leaf oil composition was unsuitable for intraspecific phylogenic studies. Here, ripe fruits of 30 accessions and 29 leaf samples belonging to three genera of *Citrus*, *Poncirus*, and *Fortunella* and 18 species in total were collected in this study, and it could be deduced that chemotaxanomic analysis based on volatile compounds in both fruit peel and leaf is suitable for interspecies phylogenetic studies.

### The loss of flavor traits in citrus cultivars compared with wild genotypes

Liu et al. [5] found that *β*-myrcene and (*Z*)- and (*E*)-linalool oxides were the characteristic aroma compounds of Mangshanyegan and (*Z*)- and (*E*)-linalool oxides had a flower, woody, green, linalool-like note [5]. In this study, it was very interesting that among 30 investigated fruit samples, (*Z*)- and (*E*)-linalool oxides were only identified in Daoxian wild mandarin, Jiangyong wild mandarin, Mangshanyegan (sharp-leaf & round-leaf), Clementine tangerine and Liangping pummelo. Also, (*Z*)- and (*E*)-linalool oxides were at trace levels in both Clementine tangerine and Liangping pummelo. Among the 14 fruit samples of Sinocitrus (samples with underline in Table 1), Daoxian wild mandarin, Jiangyong wild mandarin, Mangshanyegan (sharp-leaf & round-leaf) were wild genotypes and the others were all commercial cultivars. It was demonstrated that (*Z*)- and (*E*)-linalool oxides were not detected in the cultivated loose-skin mandarins except Clementine (trace level). The loss of flavor traits in cultivars might be attributed to breeding and selection that favors yield, disease resistance, and pleasant fruit appearance and in which flavor and aroma have been ignored for a long period of time. As a result, some superior flavor traits of wild genotypes were gradually lost.

## Supporting Information

Figure S1 **Total ion current chromatograms (TIC) (A, B, C) and the mass spectra (D, E).** (A) The global TIC of Chazhigan mandarin. (B) The TIC of dimethyl anthranilate (a part of A). (C) The TIC of the authentic standard of dimethyl anthranilate. (D) Mass spectrum of dimethyl anthranilate in B. (E) Mass spectrum of dimethyl anthranilate in C. time, retention time; m/z, mass-to-charge ratio.
(TIF)

Table S1 **The corrected peak areas of target volatile compounds detected in fruit peels of whole sample set.**
(XLS)

Table S2 **The aligned data obtained from Met-align.**
(XLS)

## References

[1] Fowler C, Hodgkin T (2004) Plant genetic resources for food and agriculture: assessing global availability. Annu Rev Environ Resour 29: 143–179.

[2] Deng XX, Hu CG, Huo H, Gou WW, Yi H (2000) A preliminary study of citrus germplasm conservation and its evaluation by RAPD analysis. Acta Hortic (ISHS) 535: 99–106 Available: http://www.actahort.org/books/535/535_10.htm. Accessed 2013 Feb 19.

[3] Schmidt MR, Wei W (2006) Loss of agro-biodiversity, uncertainty, and perceived control: a comparative risk perception study in Austria and China. Risk Anal 26: 455–470. 16573633 10.1111/j.1539-6924.2006.00744.x

[4] Li RT (2000) Investigation on botanical characters of wild mandarins from Hunan Province. Hunan Agric Sci 5: 30–31 (in Chinese).

[5] Liu C, Cheng Y, Zhang H, Deng X, Chen F, et al (2012) Volatile constituents of wild citrus Mangshanyegan (*Citrus nobilis* Lauriro) peel oil. J Agric Food Chem 60: 2617–2628. 22352344 10.1021/jf2039197

[6] Katajamaa M, Oresic M (2005) Processing methods for differential analysis of LC/MS profile data. BMC Bioinf 6: 179. 10.1186/1471-2105-6-179 PMC1187873 16026613

[7] Baran R, Kochi H, Saito N, Suematsu M, Soga T, et al (2006) MathDAMP: a package for differential analysis of metabolite profiles. BMC Bioinf 7: 530. 10.1186/1471-2105-7-530 PMC1764210 17166258

[8] Luedemann A, Strassburg K, Erban A, Kopka J (2008) TagFinder for the quantitative analysis of gas chromatography-mass spectrometry (GC-MS)-based metabolite profiling experiments. Bioinformatics 24: 732–737. 18204057 10.1093/bioinformatics/btn023

[9] Lommen A (2009) MetAlign: interface-driven, versatile metabolomics tool for hyphenated full-scan mass spectrometry data preprocessing. Anal Chem 81: 3079–3086. 19301908 10.1021/ac900036d

[10] Tikunov Y, Lommen A, de Vos CH, Verhoeven HA, Bino RJ, et al (2005) A novel approach for nontargeted data analysis for metabolomics. Large-scale profiling of tomato fruit volatiles. Plant Physiol 139: 1125–1137. 16286451 10.1104/pp.105.068130 PMC1283752

[11] Skogerson K, Wohlgemuth G, Barupal DK, Fiehn O (2011) The volatile compound BinBase mass spectral database. BMC Bioinf 12: 321. 10.1186/1471-2105-12-321 PMC3199763 21816034

[12] Aliferis KA, Cubeta MA, Jabaji S (2011) Chemotaxonomy of fungi in the *Rhizoctonia solani* species complex performing GC/MS metabolite profiling. Metabolomics Available: http://dx.doi.org/10.1007/s11306-011-0340-1. Accessed 2013 Feb 19.

[13] Fraga BM (2012) Phytochemistry and chemotaxonomy of *Sideritis* species from the Mediterranean region. Phytochemistry 76: 7–24. 22326508 10.1016/j.phytochem.2012.01.018

[14] Radulovic N, Dekic M, Joksovic M, Vukicevic R (2012) Chemotaxonomy of Serbian *Teucrium* species inferred from essential oil chemical composition: the case of *Teucrium scordium* L. ssp. *scordioides* . Chem Biodivers 9: 106–122. 22253108 10.1002/cbdv.201100204

[15] Moore GA (2001) Oranges and lemons: clues to the taxonomy of *Citrus* from molecular markers. Trends Genet 17: 536–540. 11525837 10.1016/s0168-9525(01)02442-8

[16] Kesterson JW, Pieringer AP, Edwards GJ. Hendrickson R (1964) Application of gas-liquid chromatography to the citrus leaf oils for the identification of kinds of citrus. Proc Am Soc Hortic Sci 84: 199–203.

[17] Pieringer AP, Edwards GJ, Wolford RW (1964) The identification of citrus species and varieties by instrumental analysis of citrus leaf oils. Proc Am Soc Hortic Sci 84: 204–212.

[18] Merle H, Moron M, Blazquez MA, Boira H (2004) Taxonomical contribution of essential oils in mandarins cultivars. Biochem Syst Ecol 32: 491–497.

[19] Lin Z, Hua Y (1992) Systematic evolution relation of chemical components of the essential oils from 11 taxa of citrus leaves. Acta Bot Sin 34: 133–139.

[20] Gonzalez-Mas MC, Rambla JL, Alamar MC, Gutierrez A, Granell A (2011) Comparative analysis of the volatile fraction of fruit juice from different citrus species. PLoS One 6: e22016. 21818287 10.1371/journal.pone.0022016 PMC3139606

[21] Luro F, Venturini N, Costantino G, Paolini J, Ollitrault P, et al (2012) Genetic and chemical diversity of citron (*Citrus medica* L.) based on nuclear and cytoplasmic markers and leaf essential oil composition. Phytochemistry 77: 186–196. 22264998 10.1016/j.phytochem.2011.12.013

[22] Wayman KA, de Lange PJ, Larsen L, Sansom CE, Perry NB (2010) Chemotaxonomy of Pseudowintera: sesquiterpene dialdehyde variants are species markers. Phytochemistry 71: 766–772. 20176388 10.1016/j.phytochem.2010.01.017

[23] Hou Y, Liang W, Zhang L, Cheng S, He F, et al (2011) Freshwater algae chemotaxonomy by high-performance liquid chromatographic (HPLC) analysis. Front Environ Sci Eng China 5: 84–91.

[24] Li R, Luo G, Meyers PA, Gu Y, Wang H, et al (2012) Leaf wax n-alkane chemotaxonomy of bamboo from a tropical rain forest in Southwest China. Plant Syst Evol 298: 731–738.

[25] Li YZ, Cheng YJ, Yi HL, Deng XX (2006) Genetic diversity in mandarin landraces and wild mandarins from China based on nuclear and chloroplast simple sequence repeat markers. J Hortic Sci Biotechnol 81: 371–378.

[26] Motamayor JC, Lachenaud P, da Silva EMJW, Loor R, Kuhn DN, et al (2008) Geographic and genetic population differentiation of the Amazonian chocolate tree (Theobroma cacao L). PLoS One 3: e3311. 18827930 10.1371/journal.pone.0003311 PMC2551746

[27] Vandendool H, Kratz PD (1963) A generalization of the retention index system including linear temperature programmed gas-liquid partition chromatography. J Chromatogr A 11: 463–471. 10.1016/s0021-9673(01)80947-x 14062605

[28] Liu C, Zhang H, Dai Z, Liu X, Liu Y, et al (2012) Volatile chemical and carotenoid profiles in watermelons [*Citrullus vulgaris* (Thunb.) Schrad (Cucurbitaceae)] with different flesh colors. Food Sci Biotechnol 21: 531–541.

[29] van den Berg RA, Hoefsloot HC, Westerhuis JA, Smilde AK, van der Werf MJ (2006) Centering, scaling, and transformations: improving the biological information content of metabolomics data. BMC Genomics 7: 142. 16762068 10.1186/1471-2164-7-142 PMC1534033

[30] Scora RW (1975) On the history and original of citrus. Bull Torrey Bot Club 102: 369–375.

[31] Tanaka T (1954) Species problem in *Citrus*: A critical study of wild and cultivated unites of citrus, based upon field studies in their native homes (Revisio Aurantiacearum IX). Tokyo: Japanese Society for the Promotion of Science. pp. 1–141.

[32] Swingle WT (1943) The botany of citrus and its wild relatives of the orange subfamily (family Rutaceae, subfamily Aurantioideae). In: Webber HJ, Batchelor LD, editors. The Citrus Industry. Berkeley: University of California Press. pp. 128–474.

[33] Merle H, Morón M, Blázquez MA, Boira H (2004) Taxonomical contribution of essential oils in mandarins cultivars. Biochem Syst Ecol 32: 491–497.

[34] Nicolosi E, Deng ZN, Gentile A, Malfa SL, Tribulato GC (2000) Citrus phylogeny and genetic origin of important species as investigated by molecular markers. Theor Appl Genet 100: 1155–1166.

[35] Cheng YJ, de Carmen VM, Meng HJ, Guo WW, Tao NG, et al (2005) A set of primers to analyze the chloroplast DNA diversity in *Citrus* and its relatives. Tree Physiol 25: 661–672. 15805086 10.1093/treephys/25.6.661

[36] Li RT, Zhang YN, Chen ML, Jiang KJ (2009) Taxonomy analysis on wild mandarins originating from Mangshan Mountain. Guangdong Agric Sci 8: 11–13 (in Chinese).

[37] Chen ML, Wei WN (1987) Studies on genetic relationship among wild loose skin mandarins originated from Hunan Province. Proceedings of the national conference of fruit crop resources. (Held in Chongqing, China, 1987, in Chinese)

[38] Li WB, Liu GF, He SW, Zhang YN (1993) Studies on the origin and evolutionary of Chinese loose skin mandarins based on isozyme analysis. Hunan Agric Sci 3: 3–6 (in Chinese).

[39] Li W, Liu G (1992) Leaf isozymes of mandarin. Proc Int Soc Citricult VII Congr 1: 217–220.

[40] Hirai M, Kajiura I (1987) Genetic analysis of leaf isozymes in citrus. Jpn J Breed 37: 377–388.

[41] Yamamoto M, Tominaga S (2003) High chromosomal variability of mandarins (*Citrus* spp.) revealed by CMA banding. Euphytica 129: 267–274.

[42] Herrero R, Asíns MJ, Pina JA, Carbonell EA, Navarro L (1996) Genetic diversity in the orange subfamily Aurantioideae II. Genetic relationships among genera and species. Theor Appl Genet 93: 1327–1334. 24162546 10.1007/BF00223466

[43] Fang D, Krueger RR, Roose ML (1998) Phylogenetic relationships among selected *Citrus* germplasm accessions revealed by Inter-simple sequence repeat (ISSR) markers. J Am Soc Hortic Sci 123: 612–617.

[44] Barkley NA, Roose ML, Krueger RR, Federici CT (2006) Assessing genetic diversity and population structure in a citrus germplasm collection utilizing simple sequence repeat markers (SSRs). Theor Appl Genet 112: 1519–1531. 16699791 10.1007/s00122-006-0255-9

[45] Pang XM, Hu CG, Deng XX (2003) Phylogenetic relationships among Citrus and its relatives as revealed by SSR markers. Acta Genet Sin 30: 81–87. 12812081

[46] Green R, Vardi A, Falun E (1986) The plastome of *Citrus*. Physical map, variation among *Citrus* cultivars and species and comparison with related genera. Theor Appl Genet 72: 170–172. 24247831 10.1007/BF00266989

[47] Luro FL, Costantino G, Terol J, Argout X, Allario T, et al (2008) Transferability of the EST-SSRs developed on Nules clementine (*Citrus clementina* Hort ex Tan) to other *Citrus* species and their effectiveness for genetic mapping. BMC Genomics 9: 287. 18558001 10.1186/1471-2164-9-287 PMC2435559

[48] Barkley NA, Roose ML, Krueger RR, Federici CT (2006) Assessing genetic diversity and population structure in a citrus germplasm collection utilizing simple sequence repeat markers (SSRs). Theor Appl Genet 112: 1519–1531. 16699791 10.1007/s00122-006-0255-9

[49] Tanaka T (1977) Fundamental discussion of Citrus classification. Stud Citrol 14: 1–6.

[50] Handa T, Ishizawa Y, Oogaki C (1986) Phylogenetic study of fraction I protein in the genus *Citrus* and its close related genera. Jpn J Genet 61: 15–24.

[51] Biswas MK, Chai L, Amar MH, Zhang X, Deng X (2011) Comparative analysis of genetic diversity in Citrus germplasm collection using AFLP, SSAP, SAMPL and SSR markers. Sci Hortic (Amsterdam, Neth) 129: 798–803.

[52] Biswas MK, Chai L, Mayer C, Xu Q, Guo W, Deng X (2012) Exploiting BAC-end sequences for the mining, characterization and utility of new short sequences repeat (SSR) markers in Citrus. Mol Biol Rep 39: 5373–5386. 22170603 10.1007/s11033-011-1338-5

