# Supplementary figures and images for: Chemotaxonomic Study of Citrus, Poncirus and Fortunella Genotypes Based on Peel Oil Volatile Compounds - Deciphering the Genetic Origin of Mangshanyegan (Citrus nobilis Lauriro)
Source: PLoS One. 2013 Mar 13;8(3):e58411. doi: 10.1371/journal.pone.0058411 (PMC3596367; doi:10.1371/journal.pone.0058411)

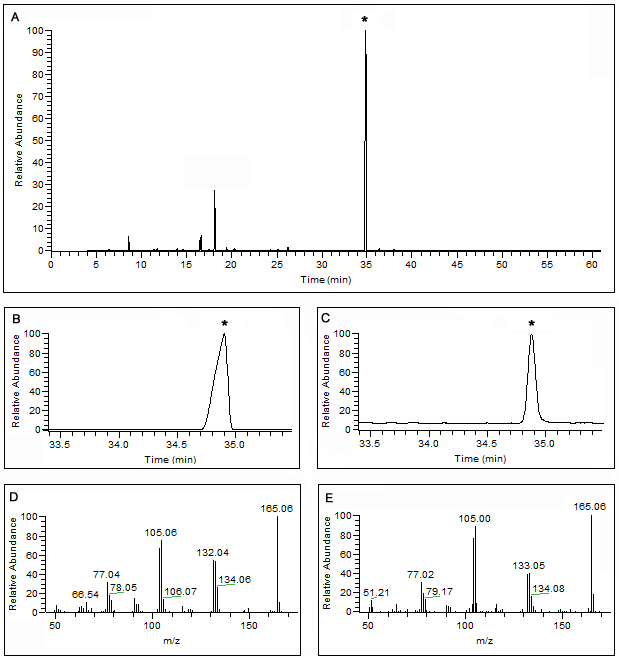

Supplement: Figure S1 — Total ion current chromatograms (TIC) (A, B, C) and the mass spectra (D, E). (A) The global TIC of Chazhigan mandarin. (B) The TIC of dimethyl anthranilate (a part of A). (C) The TIC of the authentic standard of dimethyl anthranilate. (D) Mass spectrum of dimethyl anthranilate in B. (E) Mass spectrum of dimethyl anthranilate in C. time, retention time; m/z, mass-to-charge ratio. (TIF) [file pone.0058411.s001.tif]
